# Splenic hamartomas in two children

**DOI:** 10.1186/1477-7819-12-180

**Published:** 2014-06-06

**Authors:** Li-Feng Zhang, Jin-Fa Tou, Xiang Wang, Wei-Zhong Gu, Xiao-Hui Ma, Qi Qin

**Affiliations:** 1 Department of General Surgery, Children Hospital, Zhejiang University School of Medicine, Hangzhou 310006, China; 2 Department of Pathology, Children Hospital, Zhejiang University School of Medicine, Hangzhou 310006, China; 3 Department of Radiology, Children Hospital, Zhejiang University School of Medicine, Hangzhou 310006, China

**Keywords:** Computer tomography, Immunohistochemistry, Magnetic resonance imaging, Splenic hamartoma, Ultrasonography

## Abstract

Hamartomas are extremely rare splenic benign tumours in children. We present two cases, both in boys (6 and 8 years old), with left upper quadrant abdominal pain that were otherwise asymptomatic. Both patients showed a splenic mass on preoperative ultrasonography and magnetic resonance imaging (MRI). One patient had a focal splenic mass that was identified preoperatively with contrasted computed tomography (CT) scans. Both patients underwent a total splenectomy. Although multi-modality imaging findings were described preoperatively, the final diagnosis in each case was splenic hamartoma based on histology and immunohistochemistry. The postoperative courses were uneventful.

## Background

Splenic tumours are relatively rare and include malignancies such as lymphomas, angiosarcomas, plasmacytomas, primary malignant fibrous histiocytomas, and metastatic disease. Benign splenic tumours are extremely rare and most are hemangiomas, cysts, and inflammatory pseudotumours [1,2]. Splenic hamartomas or splenomas, which were described in 1861 by Rokitansky, are extremely rare benign tumours with fewer than 150 cases having been reported in the literature [3]. The majority of these cases were found in adult patients, and only 20% of the cases were in children [4]. Although splenic hamartomas are mostly asymptomatic, particularly in adults, their association with myeloproliferative diseases and thrombocytopenia have been reported [4,5]. However, these have not been well characterised in children. Here, we report the cases of two paediatric patients with a solid lesion of the spleen, who required splenectomies and were pathologically diagnosed with splenic hamartomas.

## Case presentation

### Case 1

**Figure 1:**
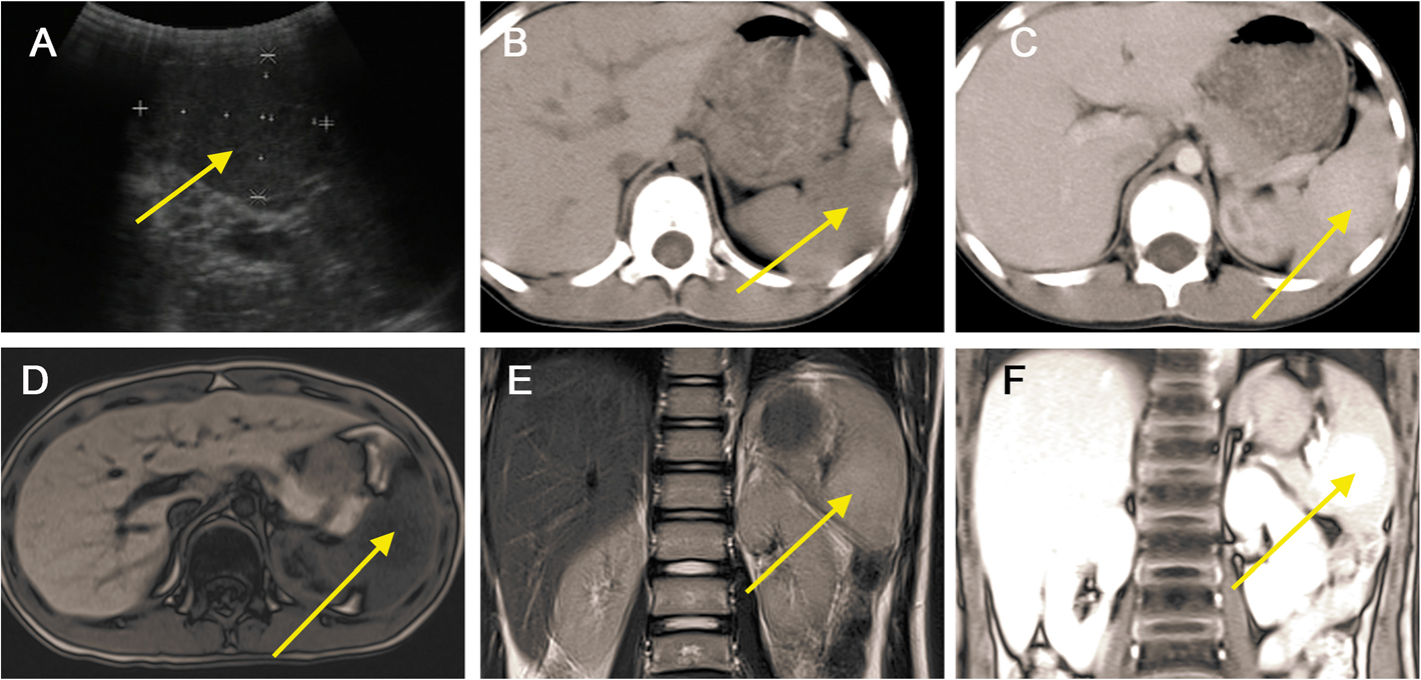
**Splenic hamartoma in an 8-year-old boy was found during examination. (A)** Abdominal ultrasonography showed an isoechoic oval-shaped mass (*arrow*) in the mid-portion of the spleen; **(B)** Non-enhanced CT showed a slightly lower-density mass (*arrow*) in the spleen. **(C)** Contrast-enhanced CT scan shows mild diffuse heterogeneous enhancement in the arterial phase. Magnetic resonance imaging (MRI) showed a hypointense mass (*arrow*) on the T1WI image **(D)** and a slightly hyperintense mass in the T2WI image **(E)**. **(F)** The lesion (*arrow*) shows moderate enhancement on gadolinium-enhanced MRI.

An 8-year-old boy was incidentally diagnosed with a splenic mass by abdominal ultrasonography and admitted to our hospital for further investigation. He had no significant past medical history. The physical examination and the laboratory findings (such as blood routine test, serum chemistry test, and tumour biomarkers) on admission were unremarkable. Abdominal ultrasonography showed an isoechoic oval-shaped mass with smooth, well-defined borders in the mid-portion of the spleen that was 5.2 cm × 4.2 cm × 3.4 cm in size, within homogeneous echo (Figure 1A). Colour Doppler showed increased internal blood flow within the mass. Non-enhanced CT showed a slightly lower-density mass with poorly-defined margins in the spleen. A contrast-enhanced CT scan showed mild diffuse heterogeneous enhancement after the intravenous administration of contrast material (Figure 1B–C). Magnetic resonance imaging (MRI) showed a hypointense mass in the T1WI image that was slightly hyperintense in the T2WI image (Figure 1D–E). The lesion showed moderate enhancement on gadolinium-enhanced MRI (Figure 1F). The differential diagnosis included hemangiomas, angiosarcomas, primary splenic lymphoma, and inflammatory lesions, such as inflammatory pseudotumour. A malignancy could not be ruled out; therefore, the patient underwent a total splenectomy. Microscopically, the nodule consisted of disorganised vascular channels lined with slightly plump endothelial cells, the muscular small blood vessels and fibrous tissue had proliferated and white pulp was visible, but was significantly reduced. On immunohistochemical staining the nodule was CD34– and CD8+, and the final diagnosis was splenic hamartoma. The platelet count began to rise slowly on the 7^th^ postoperative day and reached 1,000 × 10^9^/L on postoperative day 14. Postoperative anticoagulant prophylaxis with oral aspirin was initiated and continued for 4 weeks after surgery. Finally, the patient was discharged from the hospital on the 15^th^ postoperative day. No complications developed during postoperative follow-up, and the patient was asymptomatic 5 years after surgery.

### Case 2

**Figure 2:**
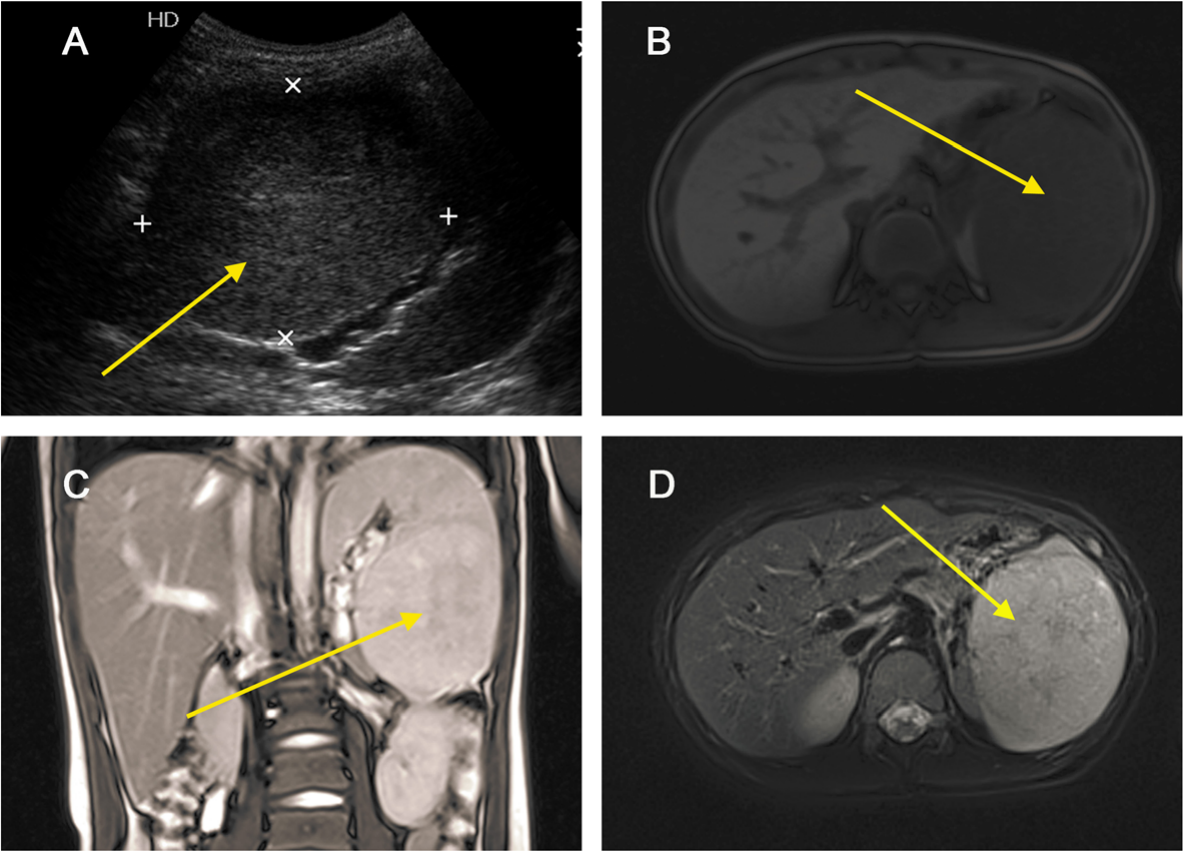
**Splenic hamartoma in a 6-year-old boy with left upper quadrant abdominal pain. (A)** Abdominal ultrasonography showed a hypoechoic mass (*arrow*) with well-defined borders in the lower pole of spleen. Magnetic resonance imaging (MRI) showed slightly hyperintense signal in the T1WI **(B)**, T2WI images **(C)**, and T2WI + Fatsat images **(D)**, and a non-homogeneous signal within the lesion (*arrow*).

**Figure 3:**
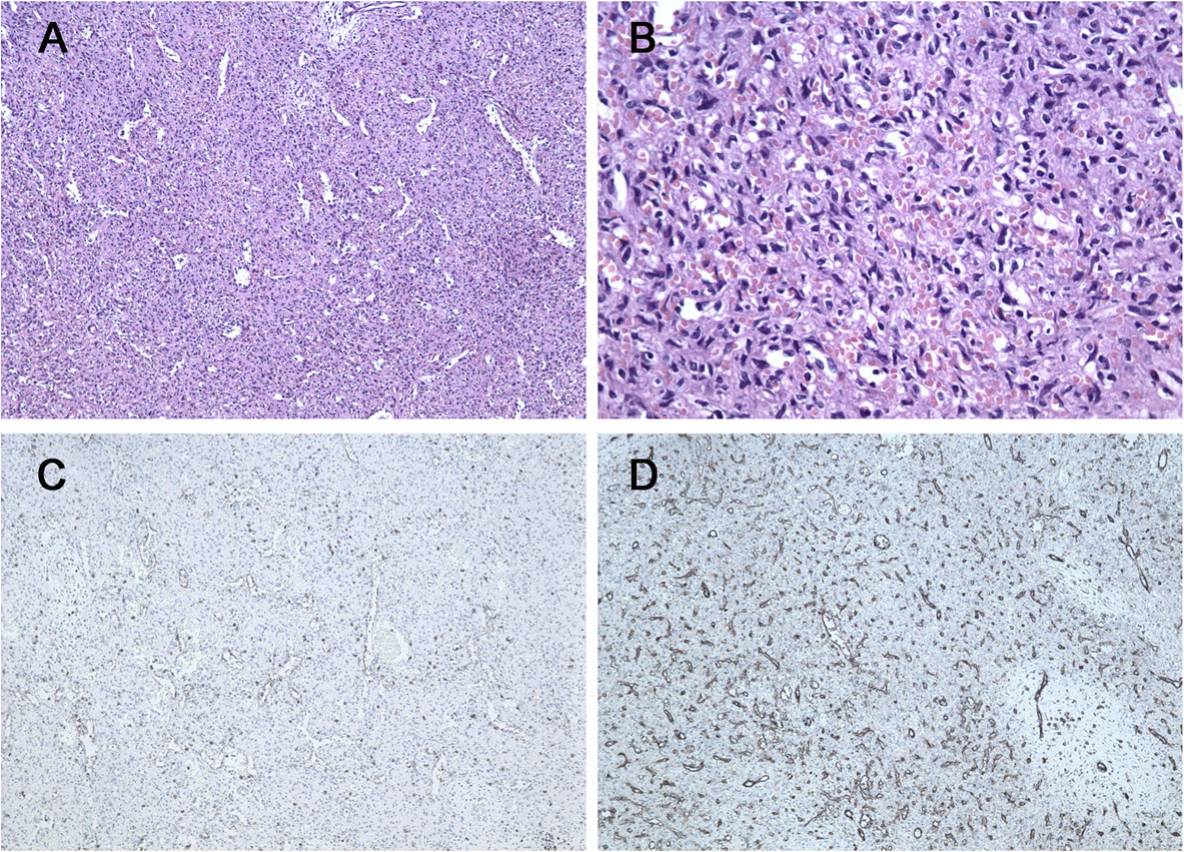
**Pathology of splenic hamartoma. (A)** Microscopic image of the splenic hamartoma showed the lesion containing a mixture of unorganised vascular channels and fibrotic cords of splenic red pulp-like area (hematoxylin-eosin, original magnification × 100). **(B)** Higher magnification of the lesion reveals no cytologic atypia and mitosis (hematoxylin-eosin, original magnification × 400). **(C)** CD8 immunostaining was positive in the lining cells and scattered lymphocytes (original magnification × 100). **(D)** Immunohistochemistry for CD31 is positive in vascular lining cells (original magnification × 100).

A 6-year-old boy was admitted to our department due to a 1-year history of left upper quadrant abdominal pain and worsening sickness for 1 week. Physical examination revealed a palpable spleen 3 cm below the costal margin. He had no significant past medical history, and the laboratory findings (such as blood routine test, serum chemistry test, and tumour biomarker) were also unremarkable. Abdominal ultrasonography showed a hypoechoic mass with well-defined borders in the lower pole of the spleen that measured 9.4 cm × 8.9 cm × 6.4 cm in size, within heterogeneous echo (Figure 2A). Colour Doppler showed increased internal blood flow within the mass. Magnetic resonance imaging (MRI) showed a slightly hyperintense signal in the T1WI and T2WI images and a non-homogeneous signal within the lesion (Figure 2B–D). The differential diagnosis included hemangiomas, angiosarcomas, and primary splenic lymphoma. Due to the risk of spontaneous rupture and the fact that malignancy could not be ruled out, the patient underwent a total splenectomy. Microscopically, the lesion contained a mixture of unorganised vascular channels and fibrotic cords within the splenic red pulp-like area (Figure 3A–B). The rest of the spleen showed unremarkable histology of red and white pulp. On immunohistochemical staining the tissue was CD8+, CD31+, and CD34–, and the final diagnosis was splenic hamartoma (Figure 3C–D). The platelet count increased to 1,108 × 10^9^/L on the 7^th^ day after surgery. Postoperative anticoagulant prophylaxis with oral aspirin was initiated and continued for 4 weeks after surgery. Finally, the patient was discharged from the hospital on the 13^th^ postoperative day. No complications developed during postoperative follow-up, and the patient was asymptomatic 17 months after surgery.

## Discussion

Splenic hamartomas are rare benign tumours with a reported incidence of 3 in 200,000 splenectomies in a single centre series [6]. The incidence of splenic hamartomas in autopsy series ranges between 0.024% and 0.13% [7].

Although most of the reports in the literature consist of adult patients [4,8], smaller reviews indicate that 20% of hamartomas occur in children, with only 30 cases having been reported in the literature [4,9-11] in addition to the two cases reported here. Overall, there were 19 males and 11 females. Nineteen of these patients had a hematologic abnormality such as anaemia, thrombocytopenia, or pancytopenia, and the specific diagnoses included bone marrow failure syndrome, sickle cell anaemia, hereditary spherocytosis, or congenital dyserythropoietic anaemia. Only 15% of the patients present with symptoms, most commonly abdominal pain, splenomegaly, cytopenia, and incidental spontaneous rupture [3,12]. However, most children present with systemic symptoms such as fever and lethargy [4,9-11].

Although the final diagnosis of splenic hamartomas is established by a pathological examination, a preoperative diagnosis using a combination of multi-modality imaging techniques may be possible [9,12-14]. On sonography, most hamartomas are hyperechoic relative to the adjacent normal splenic parenchyma [15]. Some splenic hamartomas are homogeneous and well-defined solid masses, with varying echogenicity relative to the normal splenic parenchyma, but others may be heterogeneous with cystic changes [12,16]. Colour Doppler sonography may reveal blood-flow signals within the lesions [14]. Splenic hamartomas do not always exhibit hypervascularity because some are hypoechoic and are composed of red pulp, lacking fibrous trabeculae and white pulp [16]. However, the hypoechoic lesion in patient 2 contained a mixture of unorganised vascular channels and fibrotic cords of splenic red pulp-like areas on histological examination. On unenhanced CT images, most splenic hamartomas are homogeneous, or heterogeneous low-density or isodense masses with occasional calcification [12,14]. Histopathological fibrous splenic hamartomas have a dominant fibrous tissue and MRI showed isointensity or hyperintensity on T1WI images and hypointensity on T2WI images [17]. Non-fibrous splenic hamartomas are more common in the clinic, and MRI revealed an isointense mass on T1WI images and mild hyperintense mass on T2WI images [13]. Dynamic enhanced CT and MRI are essential for suspected splenic hamartomas because of the differences from other splenic lesions. On delayed images, the density or signal of the lesion is near or slightly higher than that of the splenic parenchyma [12]. Hyperintense lesions on T2WI in both of our patients contained minimal fibrous tissue on histological examination. Therefore, sonography is a more sensitive modality than CT and MRI, which is useful for screening. CT and MRI can display components of the tumour, which are helpful for qualitative diagnosis.

Splenic hamartomas should be differentiated from the more common neoplastic disorders of the spleen such as hemangiomas and ominous lesions of the spleen including primary haemangiosarcomas, lymphomas, and metastases [12]. The main pathologic differential diagnosis is with benign vascular tumours or hemangiomas and immunohistochemical staining is required to confirm the diagnosis [4,18]. Hamartomas represent an anomalous cluster of normal splenic red pulp elements. They contain a mixture of unorganised vascular channels lined by endothelial cells and are surrounded by fibrotic cords of predominant splenic red pulp with or without (lymphoid) white pulp [19]. Because of their origin from splenic sinusoids, endothelial cells of hamartomas are CD8+ and CD34– [20]. This staining pattern also differentiates them from splenic hemangiomas, which contain CD8– and CD34+ endothelial cells.

## Conclusions

In conclusion, although splenic hamartomas are very rare tumours, they must be considered in the differential diagnosis of splenic lesions in children. However, a splenectomy may be necessary when malignancy cannot be ruled out preoperatively.

## Consent

Written informed consent was obtained from the patients for publication of this report and any accompanying images.

## Competing interests

The authors declare that they have no competing interests.

## Authors’ contributions

LFZ and QQ drafted the manuscript. JFT, WX, WZG, and XHM also assisted with manuscript preparation. QQ revised the manuscript. All authors have read and approved the final manuscript.
